# Death and suicide among former child and adolescent psychiatric patients

**DOI:** 10.1186/1471-244X-6-51

**Published:** 2006-11-02

**Authors:** Ulf Engqvist, Per-Anders Rydelius

**Affiliations:** 1 Department of Women and Child Health, Karolinska Institutet, Astrid Lindgren Children's Hospital at Karolinska University Hospital, SE-17176 Stockholm, Sweden; 2 Department of Social Work, Mid Sweden University, SE-831 25 Östersund, Sweden; 3 Department of Women and Child Health, Karolinska Institutet, Astrid Lindgren Children's Hospital at Karolinska University Hospital, SE-17176 Stockholm, Sweden

## Abstract

**Background:**

Increased mortality rates among previous child and adolescent psychiatry (CAP) patients have been found in Scandinavian studies up to the 1980s. The suicide risk in this group has been estimated to be almost five times higher than expected. This article addresses two questions: Do Swedish CAP patients continue to risk premature death and what kind of information related to psychiatric symptoms and/or behavior problems can predict later suicide?

**Methods:**

Hospital files, Sweden's census databases (including immigration and emigration) and administrative databases (including the Swedish Hospital Discharge register and the Persons Convicted of Offences register), and the Cause of Death register were examined to determine the mortality rate in a group of 1,400 former CAP inpatients and outpatients over a period of 12–33 years. Observed and expected numbers of deceased were calculated with the prospective method and the standardized mortality ratio (SMR) method. The relative risk or the risk ratio (RR) is presented with 95% confidence intervals (CIs). Significance level tests were made using two-by-two tables and chi-square tests. The Cox proportional-hazards regression model was used for survival analysis.

**Results:**

Twenty-four males and 14 females died. Compared with the general population, the standardized mortality ratio in this group of CAP patients was significantly higher in both sexes. Behavioral problems, school problems, and co-morbid alcohol or drug abuse and criminality (including alcohol-related crimes) were found to be important predictors. Thirty-two deaths were attributed to suicide, intoxication, drug overdose, or accident; one patient died of an alcohol abuse-related disorder, and five patients died of natural causes. Suicide was the most common cause of death, but only 2 of these 19 cases were initially admitted for attempted suicide.

**Conclusion:**

We suggest that suicide and death prevention among CAP patients may not be a psychiatric issue *per se *but a future function of society's juvenile social-welfare investments and juvenile-delinquency prevention programs.

## Background

Second only to death by accident, suicide is currently the most common cause of death among Swedish adolescents and young adults (males and females) in the 15–25 age group [1].

In reviews and meta-analyses, Harris and Barraclough [2,3] addressed the issue of mortality and suicide in general adult psychiatric (GenP) and child and adolescent psychiatric (CAP) care. They found that Scandinavian studies, because of reliable population database maintenance, comprised 95% of the research on psychiatric populations. According to these authors, only 11 papers from six countries outside Scandinavia, published from the 1980s onward, investigated CAP patient mortality. Overall reported suicide risk for CAP patients was almost five times that expected in the general population. Ninety-six percent of the expected value was based on statistics reported in Scandinavian studies; 65% on statistics in Swedish studies [2,3]. But the question remains: which CAP patients comprise this high-risk group? For developed Western societies, it is essential to develop strategies for suicide prevention to improve the quality of life in the general population. To do so, better knowledge is needed regarding the group of children and adolescents at the highest risk for committing suicide.

This study has two aims; First to study the rates of overall mortality and suicide among previous CAP patients and secondly to study the associations between psychosocial distress symptoms/use of psychiatric services/criminality and subsequent mortality.

## Methods

### The study group

The study group consisted of all patients who were born between 1957 and 1976 and completed their CAP treatment between 1975 and 1990. They had been admitted for inpatient or outpatient CAP treatment in Jämtland County during 1970–1990 and were then followed up until December 31, 2002. Some of the youngest patients may have been readmitted to CAP care after 1990.

Jämtland County is a sparsely populated (127,424 inhabitants) region in northern Sweden. It represents 12% of Sweden's total land mass but only 1.5% of the population. Östersund (58,459 inhabitants), the only town where psychiatric services are located, is 373 miles northwest of Stockholm.

#### Clinical services

In 1956, CAP was established in Sweden as an independent medical discipline with its own curriculum for training. According to two Parliament decisions (in 1946 and 1958), CAP was established as a free service for all Swedish children and adolescents and their families. In- and outpatient clinics and treatment homes were established under the auspices of every county council. By 1975, the organization of CAP care was fully established throughout Sweden. Jämtland County has just one CAP clinic and one general adult psychiatric clinic to serve all its inhabitants. Both are in the same hospital facility. CAP services are still free of charge.

#### CAP patients

During 1975–1990, 1,420 patients (674 young males and 746 young females) fulfilled the inclusion criteria. No records for 8 of them (4 young males and 4 young females; 6 were non-Swedes) were found in Sweden's census database, and another 12 had emigrated. So the study group included the 1,400 patients who were still living in Sweden in 2004. The mean age at first admission to CAP care was 12.1 (SD 4.1) years, mean age at completion of CAP treatment was 14.1 (SD 3.7) years, and mean age at follow-up was 34.1 (SD 4.9) years. Every fifth patient (20.4%) was an inpatient.

One-third of the outpatients were not given a formal diagnosis. Outpatients in the CAP unit did not receive diagnoses when their problems were considered temporary due to growing and maturation. Causes for admission are registered as per standards established by the Swedish Association for Child and Adolescent Psychiatry. The three most common reasons for initial CAP unit contact in the study group were behavioral symptoms, including hyperactivity (21%), relationship problems (19%), and anxious/phobic/obsessive-compulsive symptoms (13%). Attempted suicide/suicidal acts/ideation represented 5% of contacts with the CAP unit and depression represented 4%; 48% of the patients lived in a complete family (a two-parent home), and 47% had obvious problems in school. Table 1 lists reasons for admission, and Table 2 presents diagnoses as per ICD 10.

**Table 1:** The effect of different variables at baseline and during the follow up on the outcome of death and suicide.

**Independent variable**	**n**	**Dependent variable**		**Sign**	**Risk ratio**	**n**	**Dependent variable**		**Sign**	**Risk ratio**
		*Dead*	*Alive*				*Suicide*	*Alive*		

Sex	1400					1381				
Male	667	24	643	(-)		654	11	643	(-)	
Female	733	14	719			727	8	719		
										
Family	1379					1360				
Split family	724	16	708	(-)		716	8	708	(-)	
Complete family	655	22	633			644	11	633		
										
Adoption	1400					1381				
Adopted	35	2	33	(-)		34	1	33	(-)	
Natural child	1365	36	1329			1347	18	1329		
										
Problems at school	1170					1153				
Problems	544	26	518	***	1.9<4.3<9.8	529	11	518	(-)	
No problems	626	7	619			624	5	619		
										
Age at first admission to CAP	1400					1381				
Older than age 13	642	19	623	(-)		634	11	623	(-)	
13 years and younger	758	19	739			747	8	739		
										
General psychiatry care during the follow up	1400					1381				
General psychiatry care	531	23	508	**	1.3<2.5<4.8	521	13	508	**	1.3<3.5<9.2
No general psychiatry care	869	15	854			860	6	854		
										
Conviction for offenses during the follow up	1400					1381				
Convicted for offenses	499	21	478	*	1.9<2.2<4.2	486	8	478	(-)	
Not convicted for offences	901	17	884			895	11	884		
										
Reason for admission to CAP	1389					1370				
										
Anxiety	181	1	180	(-)		180	0	180	(-)	
Other cause for admission	1208	37	1171			1190	19	1171		
										
Depression	60	0	60	(-)		60	0	60	(-)	
Other cause for admission	1329	38	1291			1310	19	1291		
										
Suicide attempt, suicide thoughts	71	2	69	(-)		71	2	69	(-)	
Other cause for admission	1318	36	1282			1299	17	1282		
										
Psychosis	21	1	20	(-)		20	0	20	(-)	
Other cause for admission	1368	37	1331			1350	19	1331		
										
Behavioral disorder	292	17	275	**	1.6<3.0<5.7	282	7	275	(-)	
Other cause for admission	1097	21	1076			1088	12	1076		
										
Mental retardation and developmental problems	80	1	79	(-)		79	0	79	(-)	
Other cause for admission	1309	37	1272			1291	19	1272		
										
Abused and/or neglected	38	1	37	(-)		38	1	37	(-)	
Other cause for admission	1351	37	1314			1332	18	1314		
										
Relationship problems	258	6	252	(-)		257	5	252	(-)	
Other cause for admission	1131	32	1099			1113	14	1099		
										
Reaction to stress	52	0	52	(-)		52	0	52	(-)	
Other cause for admission	1337	38	1299			1318	19	1299		
										
Somatic problems and eating disorders	138	0	138	(-)		138	0	138	(-)	
Other cause for admission	1251	38	1213			1232	19	1213		
										
Enuresis or encopresis	47	2	45	(-)		46	1	45	(-)	
Other cause for admission	1342	36	1306			1324	18	1306		
										
Sleep disorders	34	1	33	(-)		33	0	33	(-)	
Other cause for admission	1355	37	1318			1337	19	1318		

*Notes*: *** = *P *< 0.001; ** = *P *< 0.01; * = *P *< 0.05

**Table 2:** ICD 10 diagnoses at first admission to CAP (N = 901).

***Diagnosis***	Number	Percent
Z00-Z99 Factors influencing health status and contact with health services	321	35.6
F90-F98 Behavioral and emotional disorders with onset usually occurring in childhood and adolescence	196	21.8
F40-F48 Neurotic, stress-related and somatoform disorders	140	15.5
X60-X84 Intentional self-harm	48	5.3
F50-F59 Behavioral syndromes associated with physiological disturbances and physical factors	46	5.1
F80-F89 Disorders of psychological development	39	4.3
F30-F39 Mood [affective] disorders	31	3.4
F70-F79 Mental retardation	27	3.0
F10-F19 Mental and behavioral disorders due to psychoactive substance use	24	2.7
F20-F29 Schizophrenia, schizotypal and delusional disorders	20	2.2
F60-F69 Disorders of adult personality and behavior	4	0.4
G00-G99 Diseases of the nervous system	2	0.2
E00-E90 Endocrine, nutritional and metabolic diseases	2	0.2
F99 Unspecified mental disorder	1	0.1

*Note: *ICD 10 = International Statistical Classification of Diseases and Related Health Problems 10th Revision

### Data collection

The ethics committees of Umeå University (UM document no. 95-051; UM document no. 99-023) and Karolinska Institutet (KI registration no. 99-209) granted ethical approval. A longitudinal, prospective follow-up was conducted until the end of 2003 using Swedish census and administrative records to examine mortality, use of psychiatric services, crime, and alcoholism. Nearly all (99.4%) of the patients were successfully traced (including the 12 who emigrated), but 8 were not listed in Swedish databases. CAP hospital records were then analyzed and correlated with data on death certificates and in adult psychiatry and criminal records.

The observed number of deaths and the causes of death were obtained from the national Cause of Death register (Sweden's National Board of Health and Welfare and Statistics Sweden [SCB]). Regional differences appear in the Swedish suicide rate. From 1980 to 1996, suicide rates were higher in Swedish metropolitan areas and in some regions including Jämtland County, [4] compared to other parts of Sweden. Causes of death were classified as per the English version of the International Statistical Classification of Diseases and Related Health Problems (ICD). Until 1987, ICD 8 was used in Sweden. ICD 9 was used from 1987 to 1996, and ICD 10 was introduced in l997. Diagnoses from ICD 8 and ICD 9 were re-assessed as per ICD 10, which is described in detail at: [5]

### Variables used in the statistical calculations

The dependent variables are *deceased *or *alive *and *suicide *or *alive *(see Table 1). Various independent variables are used in the statistical calculations. At baseline, the time of admission to the CAP unit, a number of independent variables were noted: sex, age, family circumstances (If the patient's biological parents were living together, this was described as a *complete family *and if not, as a *split family*), adoption and information about problems at school. The reason for admission to CAP was also noted at baseline.

Information about GenP care was available from *out- and inpatient care *records in Jämtland County but only from inpatient GenP care in the rest of Sweden. Time for first admission and diagnosis were noted.

Information about the number of patients who were registered for criminality during the follow up was based on data from the Register of Persons Convicted of Offenses. It was noted if (1) the person was found guilty in a county court; (2) had received a fine issued by a prosecutor; and/or (3) had received a waiver of prosecution issued by a prosecutor.

### Information given in the results section

In the Results section information is given about the number of deaths, causes for death, sex differences, mean age at death, expected mortality and SMR. Associations between certain distress symptoms/behavioral problems and subsequent mortality are described as well as information on GenP care, death close to or during psychiatric care (both CAP and GenP care) and criminality.

### Statistical methods

Observed and expected numbers of deceased were calculated using the prospective method described by Hartz et al. [6] and the standardized mortality ratio (SMR) method. The difference between observed and expected numbers of deceased was tested using the z test variable, [7] which we also applied in the SMR method:

z=D−EE
 MathType@MTEF@5@5@+=feaafiart1ev1aaatCvAUfKttLearuWrP9MDH5MBPbIqV92AaeXatLxBI9gBaebbnrfifHhDYfgasaacH8akY=wiFfYdH8Gipec8Eeeu0xXdbba9frFj0=OqFfea0dXdd9vqai=hGuQ8kuc9pgc9s8qqaq=dirpe0xb9q8qiLsFr0=vr0=vr0dc8meaabaqaciaacaGaaeqabaqabeGadaaakeaacqWG6bGEcqGH9aqpdaWcaaqaaiabdseaejabgkHiTiabdweafbqaamaakaaabaGaemyrauealeqaaaaaaaa@337E@

where D denotes number of observed dead, Edenotesexpected number of dead, and z the test variable is asymptotic normally distributed (0.1). If the absolute value of z is larger than 1.96, then the hypothesis of equal mortality is rejected (the 5-percentage level). The limit for the 1-percentage level is 2.58, and for the 0.1-percentage level, 3.29. All-causes SMR and sex-specific SMRs were calculated for the entire country and for Jämtland County.

The relative risk or the risk ratio (RR) is presented with 95% confidence intervals (CIs). Significance level tests were calculated using two-by-two tables and chi-square tests.

The Cox proportional-hazards regression model was used for survival analysis. The regression model is broadly applicable and the most widely used method of survival analysis. It offers the possibility of a multivariate comparison of hazard rates. All independent variables in Table 1 were used in the analysis. Survival time is defined as (1) the interval between birth year and death or end of follow-up and as (2) the interval between diagnosis and death or end of follow-up.

## Results

### Overall mortality and suicide

#### Number of deaths, sex difference, mean age at death, expected mortality, and SMR

By the end of December 2003, 2.7% of the patients – 24 males and 14 females (1.7:1.0) – had died. Among the living, the sex distribution was 719 females and 643 males (1.1:1.0) (Pearson chi-square, *P *= 0.052). Mean age at death was 26 (SD = 7.6); ages ranged from 13 to 41. Twenty-eight of the patients (74% of the deceased) died before the age of 30 years of whom eighteen died before age 25.

The all-causes SMR for the deceased was significantly (0.1% level) higher than the SMR for all of Sweden and for Jämtland County; see Tables 3 and 4. The SMR basically compares the mortality observed in the subpopulation with the mortality that could be expected. An SMR that exceeds 100 is a ratio that exceeds the norm.

**Table 3:** All-causes Standardized Mortality Rate (SMR) with 95% confidence intervals (CIs), all Sweden.

**N**	**D**	**E**	**SMR**	**z**	**95% CI**
Total, n = 1400	38	16.11	236	5.45	167–324
Males, n = 667	24	10.61	226	4.11	145–337
Females, n = 733	14	5.50	255	3.62	139–427

*Notes: *D = observed deaths, E = expected deaths, SMR = Standardized Mortality Rate, z = the sigma value, which is a description of how far a sample or point of data is away from its mean, expressed in standard deviations.

**Table 4:** All-causes Standardized Mortality Rate (SMR) with 95% confidence intervals (CIs) in Jämtland County.

**N**	**D**	**E**	**SMR**	**z**	**95% CI**
Total, n = 1400	38	17.22	221	5.01	156–303
Males, n = 667	24	11.45	210	3.71	134–312
Females, n = 733	14	5.77	243	3.43	133–407

*Notes: *D = observed deaths, E = expected deaths, SMR = Standardized Mortality Rate, z = the sigma value, which is a description of how far a sample or point of data is away from its mean, expressed in standard deviations.

#### Causes of death

Six persons died from somatic illnesses, while the others died from unnatural causes (Table 5). Nineteen patients (11 males and 8 females) committed suicide – the single most common cause of death. In the case of a twentieth patient, an intention to commit suicide was suspected but not confirmed. Males often used violent means to commit suicide, while most of the female suicide victims died of intoxication. Seven patients – all males who had experienced childhood histories of aggressive outbursts, difficulties controlling impulses, and troublesome psychosocial situations – died in traffic accidents.

**Table 5:** Unnatural and natural causes of death (N = 38).

**Cause of death**	**Males n = 24**		**Females n = 14**		**Total N = 38**		**Sex difference**
***Unnatural causes***	23		9		32		*
Suicide		11		8		19	(-)
Intention unclear		0		1		1	(-)
Drug overdose		3		0		3	(-)
Traffic accident		7		0		7	*
Other accident		2		0		2	(-)
***Natural causes (illness)***	1		5		6		*

Notes: *** p < 0.001; ** p < 0.01; * p < 0.05

### Associations between certain distress symptoms/behavioral problems and subsequent mortality

No significant differences in age or family completeness were found between those who had died compared to those still living at follow-up.

Two variables – problems at school and behavioral disorders – were the factors in the initial contacts that were found to be the most important for predicting later *death *and *suicide*, irrespective of which statistical method was used (see Tables 1 and 6).

**Table 6:** Cox regression (forward likelihood ratio [LR]), significant variables in the equation, n = 1161.

	**Variable**	**Significance**	**Hazard ratio**
Step 3	Problems at school	0.031	2.623
	Behavioral disorder	0.004	2.903
	General psychiatric care	0.014	2.449

*Note: *Survival time is defined as the length of the interval between diagnosis and death or end of follow-up.

Of the eight most frequent CAP diagnoses, three were related to higher-than-expected death rates, i.e., alcohol/drug abuse (F10-F19), behavioral and emotional disorders with onset usually occurring in childhood and adolescence (F90-F98), and unspecific observations without a certain diagnosis (Z00-Z99).

Eleven of the 19 patients (58%), who committed suicide, suffered from obvious psychosocial stress related to their home environment compared to 31% of the patients who were living. The difference was significant (*P *= 0.031). Nonetheless, inpatient CAP care was more common in the patients who had died.

#### General psychiatric care

In all, 531 (38%) former CAP patients (female: male ratio 1.7:1) were later admitted to general psychiatric care during the follow-up period. Sixty-one percent (23/38) of the deceased CAP patients, compared to 37% of those still living, needed in- or outpatient GenP care as adults (*P *= 0.004). The reasons for needing GenP care did not vary between the two groups.

#### Death close to or during psychiatric care, CAP, and GenP

Eleven (29%) of the 38 deceased patients died either while under or within 1 year of CAP (2 patients) or GenP (8 patients) care; 8 were under in- or outpatient treatment. All 11 committed suicide.

One young female died at age 16 from intoxication during CAP outpatient treatment; one young male shot himself at age 18 during a police hunt after a burglary. Four patients, two males (two and five years after CAP treatment) and two females (both 13 years after CAP treatment) committed suicide during GenP inpatient treatment. Another three patients (two males and one female) committed suicide during GenP outpatient treatment five years (the female), six years, and 21 years after CAP treatment.

Five of these eleven patients had been admitted to CAP because of behavioral disorders, the others because of relationship problems (2), anxiety (1), abuse and neglect (1), and enuresis (1) or for observation (1). Nine of the eleven had been treated in CAP *and *GenP. The reasons for GenP treatment were substance abuse (3) and schizophrenia (1), mood disorder (1), neurotic disorder (1), personality disorder (1), and behavioral and emotional disorders/other diagnosis (1).

#### Time from CAP treatment to death

The time from CAP care to death is plotted in Table 7 along with the reasons for death and age at death. As expected, 50% of all deaths occurred more than 10 years after CAP treatment. Of those who committed suicide before age 25 (11 patients, 6 males and 5 females), the majority, seven patients, committed suicide more than 5 years after CAP treatment. Seventy-four percent (28/38) of all deceased died before age 30. Half (14/28) committed suicide.

**Table 7:** Time from psychiatric care to death, cause of death and age at death.

**Age at death (year)**	**Died during CAP care**	**Time to death after completion of CAP care**					**Total**
			
		0–1 yr	1–2 years	2–5 years	5–10 years	> 10 years	
13					T		1
16	S		T				2
18		S		T	S	S	4
19				S, T			2
20				A, S, T			3
21					S		1
23					S (3)	I	4
24						S	1
25					I, S (2)		3
26						I, T	2
28						S, U	2
29						I, N, T	3
30						S	1
32						S	1
33						N, I	2
37						I, S	2
38						A	1
39						S	1
40						N	1
41						S	1
Total	1	1	1	6	9	20	38

*Notes: *A = Accident, I = Illness, N = Narcotic overdose, S = Suicide, T = Traffic accident, U = Intention unclear

#### Those who died before age 25

Eighteen patients, 13 males and five females, died before age 25. Six of them died from accidents (five in traffic accidents). One died from a somatic illness, while the remaining eleven committed suicide. Eight (44%) had a criminal record. Only one (a young male) had been admitted to CAP due to a suicide attempt, while 13 had been admitted for behavioral and relationship problems or because the authorities needed the patients' testimony (4). One patient was admitted for anxiety, enuresis and encopresis, sleep problems, and observation.

#### Death and criminality

Of the former CAP patients, 36% (i.e., 499 out of 1,400 or every second male and every fifth female) were entered in the Persons Convicted of Offences register during the follow-up period. The patients who died during those years committed many crimes (*P *= 0.010).

Of the deceased, more patients were registered for repeated (>five times) criminality (*P *= 0.000) than for none or less than six criminal offenses. Drug-related criminality was also more prevalent among the deceased.

## Discussion

### Overall mortality and suicide

These results show that a group of Swedish CAP patients, especially those with behavioral problems, currently run an elevated risk of early death, despite overall improvement in health that has occurred in the Swedish population in recent decades. Twenty-eight of the patients (74% of the deceased) died before age 30. The risk of dying was almost twice as high for young males as for young females. Thirteen males and five females died before age 25. Only one (a young male) had been admitted for CAP treatment because of a suicide attempt. In this group, behavioral and relationship problems were the most common reasons for referral for CAP treatment. None of them had been under treatment for schizophrenia or bipolar disorder and almost half (44%) had a criminal record.

Nineteen (50%) of the 38 former patients who died committed suicide. Together with another patient, where suicidal intention was unclear, this entails an approximate suicidal death rate of 14.3 per 1,000 persons in the cohort of former CAP patients. Although suicide was the most common cause of death, only 2 of the 19, who later committed suicide, had been initially admitted for CAP clinical care because of attempted suicide. Eleven of these 19 individuals exhibited obvious psychosocial risk factors related to their home environments at their first admission to a CAP unit. Eleven patients committed suicide within one year of their last psychiatric treatment (CAP or GenP). Only two of these had been under treatment for schizophrenia (1) and mood disorder (1). Behavioral problems, problems at school, and crime were common, irrespective of the cause of death, whereas suicide attempts constituted a poor predictor for later suicides.

The findings of this study are in line with results from previous studies of Swedish CAP patients. In 1928, Alice Hellström, a Swedish pioneer in child and adolescent psychiatry, initiated a Swedish CAP longitudinal study of behaviorally disturbed children [8]. Up to 1968, the study followed 242 children (154 young males and 88 young females) who had undergone treatment between 1928 and 1940. Ten percent of this cohort (16 young males and 8 young females) died during the follow-up period. Four percent of the cohort (10 young males and 6 young females) died before age 30. Of the 10 young males, four died from disease, four from accident, and one (age 18) from suicide. Of the six young females, four died from disease and two from suicide (ages 26 and 27).

In the 1950s, a study of 2,164 children was launched; these children were treated via outpatient CAP services in Stockholm. Subgroups of 222 Stockholm schoolboys (a randomized sample from the general population) and 100 delinquent young males from the same age groups (born 1939–1953) were described using the same criteria and followed for 20 years and 18 years, respectively. Of the 2,164 former CAP patients, an overall mortality rate of 2.1% (2.6% for males, 1.3% for females) was found. One-third of the deaths were suicides, and one-third of the deaths were caused by accidents or alcohol-related illnesses [9]. Among the 222 average schoolboys, 8 (3.6%) died during the 18-year follow-up (6 died from diseases and 2 from accidents). In the delinquent group, 9% died from accidents or suicides (of whom 44% died before age 17) [10].

In a 30-year follow-up study of the former CAP patients, de Chateau reported a 4.8% mortality rate and 1.5% suicide mortality rate. The death rate was twice as high as the expected death rate in a reference group of Stockholm males and females of the same age distribution [11]. In 1984, Rydelius [12] surveyed 1,206 CAP patients who underwent treatment between 1970 and 1980 in a Stockholm hospital. Two percent had died by the end of 1981. Similar results were found in Norway [13], Denmark [14-16], and Finland [17].

### Associations between certain distress symptoms/behavioral problems and subsequent mortality

These results from Swedish prospective studies indicated a possible link between psychosocial background factors and early death due to accident and suicide [12]. A hypothesis regarding such a link between psychosocial risk factors, delinquency, and sudden violent death, including suicide, was then tested in a 19-year follow-up study of 1,064 Swedish juvenile delinquents. The results supported the hypothesis and demonstrated that 13% of the delinquent young males and 10% of the young females had died, mainly from suicide and accidents [18].

Suicide attempts are common among Swedish CAP emergency patients in metropolitan areas [19]. In 1973, nearly every fourth emergency patient in Stockholm (32% of the young females and 7% of the young males) was referred for treatment for attempted suicide. In 1995, 16% of all CAP emergency cases in Stockholm were admitted because of attempted suicide (21% of the young females and 7% of the young males). But despite the high rate of attempted suicides in Swedish CAP clinical practice, Otto [20] found a low risk of successfully completed suicides in the emergency patient group in his 15-year follow-up study.

The suicide risk for CAP patients was found to be almost five times higher than expected in the general population according to a meta-analysis and reviews [2,3]. The results of prospective follow-up studies into the 1990s of different cohorts of Swedish CAP patients, children from the general population, and delinquent children and adolescents (observation time 15–40 years) indicate that delinquent children and adolescents have the highest risk of death, including suicide.

From the results of this study, it seems reasonable to conclude that problems in school, behavioral symptoms, and conduct symptoms are more important in the calculation of risk of early death or suicide than are suicidal attempts. These findings are important for understanding the mortality risk in CAP clinical practice and for society's programs for social support to children and youth. The results are in line with Hawton's comments from 2005:"A history of behavioural disturbance, substance misuse, and family, social, and psychological problems is common" [21]. However, the findings are not in line with the recent statement by Brent at al from 2006 [22] saying: "However, the single biggest risk factor for completed suicide is a previous suicide attempt, even after controlling for psychiatric disorder". The long history of longitudinal and prospective follow-ups of different Swedish CAP cohorts over the past 80 years do point to important differences when assessing risk factors for future suicides among children and youth in CAP care compared to adults in GenP care.

Our present findings are also in line with results from a recent Swedish study on predictors of suicide from the Swedish Pregnancy and Birth registers, which indicated that psychosocial factors (low maternal education, teenage motherhood) and factors relating to the pregnancies (multiparity, restricted fetal growth) were associated with suicide completion and suicide attempts among offspring [23].

In turn, the results brought into focus the question of how prevention of suicide in children and adolescents should be managed. Today, few evidence-based suicide prevention programs or strategies have passed the scrutiny of meta-analysis [24]. The results of this study and previous Swedish studies indicate that prevention based on psychiatric measures alone is probably ineffective. In our opinion, strategies for prevention should be developed in close cooperation among healthcare, social services, and school authorities. In a review of studies that surveyed diagnoses of mental disorders in cases of completed suicide – with or without a history of admission to a mental hospital – Bertolote et al. stated (in line with our findings) that the mental health paradigm in suicide prevention, with anti-suicide strategies that focus exclusively on identification and treatment of depression, should be reconsidered. More emphasis should be placed on psychosocial and environmental interventions that diminish and counteract stress [25].

But to do this, collaboration between social work, CAP, and GenP must be developed extensively [20], because for this group of high-risk children and adolescents "providing continuity of care is a challenge, because they are often noncompliant and commonly drop out or prematurely terminate their treatment" [26].

## Limitations

The study group described above included CAP patients from a small county in mid-northern Sweden. Östersund, the only city in this county, is a university town; a Swedish military center; and an average, modern Swedish city. Although a comparison with CAP inpatients in the Stockholm metropolitan area revealed few significant between-group differences, it should be kept in mind that the study group came from a sparsely populated region. The input data were based on information from local hospital files and hospital databases at the Jämtland County Council, while outcome data were based on local registers and nation-wide databases managed by the National Board of Health and Welfare, crime authorities, and Statistics Sweden.

Suicide attempts as causes of admission to CAP care were systematically recorded in the hospital files. The CAP and GenP hospital records for all those who committed suicide and those who died in an accident were systematically scrutinized. As shown in table 1, 71 individuals were admitted to CAP due to suicide attempts/contemplating suicide. Only 2 of these 71 individuals were found dead during the follow up, in both cases due to suicide. In many respects, hospital records should not always be regarded as scientifically correct examination instruments. Because reasons for admission are used in the multivariate analyses, rather than diagnoses, comparisons with other studies may be more difficult.

The Swedish Causes of Death Register is based upon death certificates signed by physicians. If death is caused by an already diagnosed somatic disease/disorder, the certificate is either based on a clinical examination and the physician's files or on a clinical autopsy. If death is unexpected, "sudden and or violent" and is not related to hospital care, the certificate is based on a forensic autopsy. In some cases and despite a full forensic autopsy the cause of death can not be established. In these cases the term "uncertain cause of death" is used. The sample size of >1400 CAP patients and an observation time ranging from 13–28 years of length, are adequate for analyzing early deaths in this cohort, despite the fact that the results showed that the number of deaths was relatively small.

No personal contact existed between the patients and the authors.

## Conclusion

Death rates among Swedish children and adolescents in general have declined over the past 100 years. Comparing the current findings to the results from a previous longitudinal prospective follow-up of Swedish CAP – patients (following them over 30 years, from the 1950's to the 1980's [11], our findings indicate that there is still an elevated risk in the present CAP cohort. Only 2 of the 19 patients who later committed suicide were initially admitted to CAP care for attempted suicide. The results suggest that in CAP practice, variables, such as childhood psychosocial risk factors and social maladjustment symptoms, may be the most important predictors of early death, including suicide. If further studies support this hypothesis, then current opinion on preventing suicide among children and adolescents from psychiatric measures alone must change and include strong cooperation with social services and school authorities.

## Competing interests

The author(s) declare that they have no competing interests.

## Authors' contributions

UE collected the data, performed the statistical analysis, and drafted the manuscript. PAR participated in the study's design and drafted the manuscript. Both authors read and approved the final manuscript.

## Pre-publication history

The pre-publication history for this paper can be accessed here:


